# Lipid management in the prevention of stroke: a meta-analysis of fibrates for stroke prevention

**DOI:** 10.1186/1471-2377-13-1

**Published:** 2013-01-03

**Authors:** Yu-Hao Zhou, Xiao-Fei Ye, Fei-Fei Yu, Xiao Zhang, Ying-Yi Qin, Jian Lu, Jia He

**Affiliations:** 1 Department of Health Statistics, Second Military Medical University, Shanghai, 200433, China

**Keywords:** Fibrates, Stroke, Meta-analysis

## Abstract

**Background:**

Fibrates has been extensively used to improve plasma lipid levels and prevent adverse cardiovascular outcomes. However, the effect of fibrates on stroke is unclear at the present time. We therefore carried out a comprehensive systematic review and meta-analysis to evaluate the effects of fibrates on stroke.

**Methods:**

We systematically searched Medline, Embase, the Cochrane Central Register of Controlled Trials, reference lists of articles, and proceedings of major meetings to identify studies for our analysis. We included randomized placebo controlled trials which reported the effects of fibrates on stroke. Relative risk (RR) was used to measure the effect of fibrates on the risk of stroke under random effect model. The analysis was further stratified by factors that could affect the treatment effects.

**Results:**

Overall, fibrate therapy was not associated with a significant reduction on the risk of stroke (RR, 1.02, 95% CI, 0.90 to 1.16, P = 0.78). In the subgroup analyses, we observed that gemfibrozil therapy showed a beneficial effect on stroke (RR, 0.72, 95% CI, 0.53 to 0.98, P = 0.04). Similarly, fibrate therapy comparing to placebo had no effect on the incidence of fatal stroke. Subgroup analysis suggested that fibrate therapy showed an effect on fatal stroke when the Jadad score more than 3 (RR, 0.41, 95% CI, 0.17 to 1.00, P = 0.049). Furthermore, a sensitivity analysis indicated that fibrate therapy may play a role in fatal stroke (RR, 0.49, 95% CI, 0.26 to 0.93, P = 0.03) for patients with previous diabetes, cardiovascular disease or stroke.

**Conclusions:**

Our study indicated that fibrate therapy might play an important role in reducing the risk of fatal stroke in patients with previous diabetes, cardiovascular disease or stroke. However, it did not have an effect on the incidence of stroke.

## Background

Cardiovascular disease is the leading cause of premature morbidity and mortality for both men and women worldwide, accounting for 30.9% of global mortality and 10.3% of global burden of disease [1,2]. Over the past few decades, a series of studies have shown a strong correlation between hypertriglyceridemia and cardiovascular disease. Those studies indicated that elevated triglyceride levels as a risk factor of coronary artery disease. In addition, it has been suggested that raised triglycerides in the blood should be lowered as a therapeutic approach to prevent cardiovascular disease [3-7]. However, reduction of the concentrations of triglycerides in the blood has not been shown consistently to be beneficial for stroke [8].

Recently, a meta-analysis [5] revealed that statins could effectively achieve target cholesterol goals and reduce the risk of stroke. However, certain patients intolerant to statins also need stroke prevention. In addition, a high residual risk of coronary and other cardiovascular events persists during the statin therapy. It is necessary to find additional effective preventive therapies. Fibrates has been clearly shown to be effective in elevating HDL cholesterol, lowering triglyceride concentrations, reducing LDL cholesterol and chylomicron remnants [9]. However, inconsistent clinical results have been reported [8,10], and the efficacy of fibrates lowering triglyceride levels in reducing the risk of stroke has not been confirmed by randomized trials.

Although a number of trials indicated that fibrates had limited effect on the event of stroke. VA-HIT study [10] showed that fibrates significantly reduced the risk of stroke. In order to reveal the effect of fibrates on the event of stroke further, data from recent trials is needed to be re-evaluated and combined with former literatures. Therefore, we carried out a systematic review and meta-analysis of pooled data from randomized controlled trials which reported stroke as the endpoint in relation to fibrate therapy.

## Methods

### Data sources, search strategy, and selection criteria

Randomized, double-blind, placebo-controlled, and trials of fibrate therapy in English-language literature were eligible for inclusion in our research, regardless of publication status (published, unpublished, in press, or in progress). References of our meta-analysis were identified through searches of Pubmed, EmBase, and the Cochrane Central Register of Controlled Trials, with a date up to Dec 10, 2011. We searched with the following terms “clofibrate”, “bezafibrate”, “gemfibrozil”, “fenofibrate”, “procetofen”, and “randomised controlled trials”. The search was restricted to trials in human beings and published in English. References were also identified by screening the proceedings of annual meeting, bibliographies of publications for potentially relevant trials. We restricted our research to randomized controlled trials, which were less likely to be subject to confounding and bias than observational studies. Studies were eligible for inclusion when they met the following requirements: randomized controlled design; the intervention duration was at least 6 months and follow-up period was more than 12 months; recorded data on the event of stroke. The literature search was undertaken independently by 2 authors (Fei-Fei Yu and Ying-Yi. Qin) with a standardized approach, and any disagreement between these 2 authors was settled by a third author (Yu-Hao. Zhou) until a consensus was reached. This review was conducted and reported according to the PRISMA (Preferred Reporting Items for Systematic Reviews and Meta-Analysis) Statement issued in 2009 (Additional file 1) [11].

### Data collection and quality assessment

3,279 identified studies were reviewed by 2 authors (Xiao-Fei Ye and Xiao Zhang) independently. Other two investigators (Ying-Yi Qin and Jian Lu) independently checked each full-text trial for eligibility and extracted and tabulated all relevant data with a standard protocol and reviewed by a third investigators (Yu-Hao Zhou). Any discrepancy was settled by group discussion, and then the primary authors (Yu-Hao. Zhou and Jia He) made the final decision. Recorded data variables were shown as follows: first author or study group’s name, publication year, the number of patients enrolled, gender, mean age, pre-existent diseases, percentage of diabetes, total cholesterol, blinding, interventions, control, the duration of follow-up, lifestyle modification, and the event of stroke. Study quality was assessed by the Jadad score [12].

### Statistical analysis

We assessed the overall effect of fibrate therapy on the risk of stroke based on all data from the included trials. Outcome was reported by relative risks (RR) with 95% confidence intervals (CIs) to evaluate the effect of fibrate therapy on the event of stroke. We then performed subgroup analysis by the type of drug (clofibrate, bezafibrate, gemfibrozil, or fenofibrate), mean age (≥60 or <60), triglyceride lowering (≥10% or <10%), pre-exsistent diseases (diabetes, stroke, or other), number of patients (≥1000 or <1000), published years (after 2000 or previous), duration of follow-up (≥60 months or <60 months), baseline total cholesterol (≥6.0 mmol/L or <6.0 mmol/L), total cholesterol lowering (≥5% or <5%), triglyceridemia (≥30% or <30%), or Jadad score (score 4 or 5, less than 4). Furthermore, the effect of between-group triglyceride lowering, and total cholesterol change on stroke incidence was assessed by linear regression model for the logarithmic relative risk of stroke. Although the fixed-effect and random-effect models yielded similar conclusions, we used random-effect model with Mantel-Haenszel statistics in our study. Under this model, we assumed that the true underlying effect varied among included trials due to the different pre-existent diseases, intervention regimens, and the duration of follow-up which were involved in the original trials. Moreover, many investigators also considered the random-effect model to be a more natural choice than the fixed-effect model in medical decision-making contexts [13,14]. Heterogeneity of treatment effects among studies was investigated by scatter plot analysis and the heterogeneity I^2^ statistic [15]. Egger’s test [16] was used to check for potential publication bias. All P values were reported as two-sided and P values less than 0.05 were regarded as statistically significant for all included studies. SAS software (version 9.1.3) was used for the regression analysis and STATA (version 10.0) was used for the meta-analysis.

## Results

We identified 3,279 potential trials from our initial searches and 3,191 were excluded during a preliminary review. Among the 88 trials retrieved for detailed assessment, 78 trials were excluded for lack of data on stroke, polytherapy in either treatment group or control group, or reporting the same population. Our final analysis included 10 randomized controlled trials, which consisted of 37,791 individual patients. Table 1 summarized the baseline characteristics of the included studies and their participants. Design characteristics of included trials were presented in Table 2. The trials compared fibrate therapy with placebo were included in our research. Sample size of the trials ranged from 95 to 10,627, with a mean of 3,779, and the duration of follow-up for patients ranged from 30 to 104 months. 4 of included trials [17-20] evaluated the effect of clofibrates, 2 trials [8,21,22] evaluated the effect of bezafibrate, 2 studies [10,23] evaluated the effect of gemfibrozil, and the remaining 2 studies [24,25] evaluated the effect of fenofibrate. In addition, 9 trials included patients with pre-existing disease: 2 trials [17,18] reported that patients had the history of cerebral or TIA, and the remaining 7 trials [8,10,19,21-25] reported patients had the history of myocardial infarction, diabetes, coronary disease or lower extremity arterial disease. Another trial [20] included participants with high level of cholesterol. The quality of the trials was assessed according to the pre-fixed criteria using the Jadad score. Among the 10 included trials, five trials [8,10,19,24,25] scored 4, one [23] scored 3, two [20,21] scored 2, one [18] scored 1 and the remaining one trial [17] scored 0.

**Table 1:** Baseline characteristic of trials participants

**Source**	**No. of patients**	**Gender (male)**	**Mean age, y**	**Pre-existent diseases**	**Diabetes**	**Total cholesterol (mmol/L)**
BIP study [8] (2000)	3090	2825 (91%)	60	MI more than 6 month, and less than 5 year and/or stable angina	10%	5.5
VA-HIT study [10] (1999)	2531	2531 (100%)	64	Histories of CHD	25%	4.5
Acheson J [17] (1972)	95	65 (68%)	NR	Cerebral vascular disease	Excluded severe diabetics	7.5
Veterans Administration Cooperative Study Group [18] (1973)	532	532 (100%)	NR	cerebral I or TIA within 12 month	24%	6.2
Coronary Drug Project Research Group [19] (1975)	3892	3892 (100%)	NR	MI more than 3 month	NR	6.5
WHO-COOP committee of principal Investigators [20] (1978)	10627	10627 (100%)	46	Upper third level of cholesterol from 15 745 healthy men	0%	6.4
LEADER study [21,22] (2002)	1568	1568 (100%)	68	lower extremity arterial disease	66%	5.6
E J Whitney [23] (2005)	9795	6138 (63%)	62	Type 2 diabetes mellitus	100%	5.0
FIELD study [24] (2005)	143	132 (92%)	63	Low HDL-C and coronary disease	NR	5.1
The ACCORD Study Group [25] (2010)	5518	3824 (69%)	62	Type 2 diabetes mellitus	100%	5.0

**Table 2:** Design of trials included in the systematic review and meta-analysis

**Source**	**Blinding**	**Intervention**	**Control**	**Follow-up (month)**	**Lifestyle intervention**	**Jadad score**
BIP study [8] (2000)	Double	Bezafibrate 400 mg daily	Placebo	74.4	Yes	4
VA-HIT study [10] (1999)	Double	Gemfibrozil 1200 mg daily	Placebo	36–60	Yes	4
Acheson J [17] (1972)	Open	Clofibrate 1–2 g daily	Corn oil then placebo	104 in treatment group 91 in placebo group	NR	0
Veterans Administration Cooperative Study Group [18] (1973)	Double	Clofibrate 2 g daily	Placebo	54	NR	1
Coronary Drug Project Research Group [19] (1975)	Double	Clofibrate 1.8 g daily	Placebo	74.4	NR	4
WHO-COOP committee of principal Investigators [20] (1978)	Double	Clofibrate 1.6 g daily	Olive oil Placebo	63.6	Yes	2
LEADER study [21,22] (2002)	Double	Bezafibrate 400 mg daily	Placebo	55.2	NR	2
E J Whitney [23] (2005)	Double	Gemfibrozil 600 mg daily	Placebo	30	Yes	3
FIELD study [24] (2005)	Double	Fenofibrate 200 mg daily	Placebo	60	Yes	4
The ACCORD Study Group [25] (2010)	Double	Fenofibrate 160 mg daily	Placebo	56.4	Yes	4

After pooling included trials, we concluded that fibrate therapy had no effect on the risk of stroke (RR, 1.02; 95% CI: 0.90 to 1.16). Heterogeneity test for all analysis in Table 3 showed that all P value for heterogeneity were larger than 0.05, and heterogeneity was not statistically significant in the overall analysis and in subgroup analysis. Furthermore, sensitivity analysis also showed that the results were not affected by sequential exclusion of any particular trial.

**Table 3:** Subgroup analysis for the effect of fibrates therapy on stoke, and fatal stroke

	**Group**	**Stroke event/total patients**		**Relative risk (RR)**	**P value**	**Heterogeneity**	**P value for heterogeneity**
		**Fibrates**	**Placebo**				
**stroke**	**Published years**						
	after 2000	341/10062	351/10052	0.97 (0.84 to 1.13)	0.72	0%	0.51
	before 2000	292/8013	471/9664	1.06 (0.84 to 1.33)	0.63	52%	0.08
	**Number of patients**						
	≥1000	573/17689	775/19332	0.99 (0.87, 1.12)	0.85	23%	0.26
	<1000	60/386	47/384	1.23 (0.80, 1.90)	0.34	30%	0.24
	**Mean age**						
	<60	32/5331	27/5296	1.18 (0.71 to 1.96)	0.53	-	-
	≥60	405/11326	439/11319	0.93 (0.80 to 1.08)	0.34	17%	0.30
	**Gender**						
	male	329/8749	498/10401	1.09 (0.86 to 1.38)	0.47	56%	0.06
	Male/female	304/9326	324/9315	0.95 (0.82 to 1.10)	0.48	0%	0.77
	**Drug**						
	clofibrate	228/6749	383/8397	1.15 (0.98 to 1.34)	0.08	0%	0.59
	bezafibrate	132/2331	126/2327	1.05 (0.80 to 1.38)	0.37	21%	0.26
	gemfibrozil	64/1335	90/1339	0.72 (0.53 to 0.98)	0.04	0%	0.41
	fenofibrate	209/7660	223/7653	0.94 (0.78 to 1.13)	0.49	0%	0.49
	**Control**						
	corn or olive oil	55/5378	49/5344	1.11 (0.80 to 1.54)	0.53	0%	0.76
	placebo	572/12697	761/14372	1.02 (0.88 to 1.17)	0.83	32%	0.17
	**Follow-up**						
	≥60 months	421/12924	612/14575	1.01 (0.90 to 1.14)	0.85	0%	0.62
	<60 months	212/5151	210/5141	1.05 (0.76 to 1.46)	0.75	59%	0.05
	**Total cholesterol**						
	≥6.0 mmol/L	228/6749	383/8397	1.15 (0.98 to 1.34)	0.08	0%	0.59
	<6.0 mmol/L	405/11326	439/11319	0.93 (0.80 to 1.08)	0.34	17%	0.30
	**Total cholesterol lowering**						
	≥5%	409/12230	586/13890	1.04 (0.92 to 1.18)	0.49	0%	0.51
	<5%	173/3080	188/3073	0.98 (0.67 to 1.45)	0.93	71%	0.03
	**Triglyceride lowering**						
	≥30%	101/1603	113/1603	0.96 (0.46 to 1.99)	0.92	74%	0.02
	<30%	477/11094	660/12769	1.02 (0.91 to 1.15)	0.69	0%	0.51
	**Pre-exsistent diseases**						
	stroke	60/315	45/312	1.28 (0.86 to 1.90)	0.23	35%	0.22
	diabetes	209/7660	223/7653	0.94 (0.78 to 1.13)	0.49	0%	0.49
	other	364/10100	554/11751	1.00 (0.83 to 1.20)	0.99	36%	0.16
	**Jadad score**						
	4	481/11575	699/13251	0.95 (0.82 to 1.09)	0.48	30%	0.22
	<4	152/6500	123/6465	1.22 (0.98 to 1.52)	0.07	0%	0.58
	**Overall**	633/18075	822/19716	1.02 (0.90 to 1.16)	0.78	27%	0.20
**Fatal stroke**	**Published years**						
	after 2000	17/3548	17/3538	0.93 (0.33 to 2.60)	0.89	51%	0.15
	before 2000	24/6910	35/6875	0.70 (0.41 to 1.19)	0.18	0%	0.47
	**Number of patients**						
	≥1000	34/10143	40/10101	0.82 (0.45 to 1.48)	0.51	32%	0.22
	<1000	7/315	12/312	0.59 (0.23 to 1.47)	0.26	0%	0.59
	**Mean age**						
	<60	14/5331	14/5296	0.99 (0.47 to 2.08)	0.99	-	-
	≥60	20/4812	26/4805	0.69 (0.27 to 1.75)	0.44	53%	0.12
	**Gender**						
	male	35/7646	39/7612	0.89 (0.53 to 1.51)	0.67	19%	0.30
	Male/female	6/2812	13/2801	0.46 (0.18 to 1.21)	0.12	0%	0.85
	**Drug**						
	clofibrate	21/5646	26/5608	0.81 (0.45 to 1.44)	0.47	0%	0.59
	bezafibrate	13/783	9/785	1.45 (0.62 to 3.37)	0.39	-	-
	gemfibrozil	3/1264	9/1267	0.33 (0.09 to 1.23)	0.10	-	-
	fenofibrate	4/2765	8/2753	0.50 (0.15 to 1.65)	0.25	-	-
	**Control**						
	corn or olive oil	16/5378	19/5344	0.85 (0.43 to 1.66)	0.63	0%	0.32
	placebo	25/5151	33/5141	0.73 (0.38 to 1.40)	0.34	30%	0.23
	**Follow-up**						
	≥60 months	16/5378	19/5344	0.85 (0.43 to 1.66)	0.63	0%	0.32
	<60 months	25/5080	33/5069	0.73 (0.38 to 1.40)	0.34	30%	0.23
	**Total cholesterol**						
	≥6.0 mmol/L	21/5646	26/5608	0.81 (0.45 to 1.44)	0.47	0%	0.59
	<6.0 mmol/L	20/4812	26/4805	0.69 (0.27 to 1.75)	0.44	53%	0.12
	**Total cholesterol lowering**						
	≥5%	29/6161	28/6129	1.04 (0.62 to 1.76)	0.87	0%	0.38
	<5%	8/1532	16/1531	0.51 (0.22 to 1.20)	0.12	0%	0.40
	**Triglyceride lowering**						
	≥30%	8/1532	16/1531	0.51 (0.22 to 1.20)	0.12	0%	0.40
	<30%	17/3548	17/3538	0.93 (0.33 to 2.60)	0.89	51%	0.15
	**Pre-exsistent diseases**						
	stroke	7/315	12/312	0.59 (0.23 to 1.47)	0.26	0%	0.59
	diabetes	4/2765	8/2753	0.50 (0.15 to 1.65)	0.25	-	-
	other	30/7378	32/7348	0.91 (0.45 to 1.83)	0.78	42%	0.18
	**Jadad score**						
	4	7/4029	17/4020	0.41 (0.17 to 1.00)	0.05	0%	0.66
	<4	34/6429	35/6393	0.97 (0.60 to 1.57)	0.91	0%	0.51
	**Overall**	41/10458	52/10413	0.79 (0.51 to 1.23)	0.30	6%	0.38

Six trials [10,17,18,20,23,25] included 20871 individuals and 93 total events of fatal stroke were recorded. There was no evidence to support that fibrate therapy protected against fatal stroke (RR, 0.79; 95% CI, 0.51 to 1.23, Table 3) with homogenity across included studies. Furthermore, we observed that the results were not affected by excluding of any specific trial from the pooled analysis.

Post-intervention total cholesterol lowering was measured in all included studies except one [23]. There was considerable variation in the net and relative reduction of total cholesterol concentration among the included trials, ranging from 3.0% to 19.6% (Table 4). We observed an inverse relation between total cholesterol lowering and incidence of stroke (r = −0.68, p = 0.044). When we stratified the trials by the degree of total cholesterol lowering, the RR in total cholesterol of less than 5% was 0.98 (95% CI, 0.67 to 1.45, P = 0.93), and the RR in total cholesterol of 5% or more was 1.04 (95%, 0.92 to 1.18, P = 0.49). Although we found inverse relation between total cholesterol lowering and incidence of stroke, the results were not affected by subgroup analysis. Similarly, there seemed to be related between total cholesterol lowering and incidence of fatal stroke, triglyceride lowering and incidence of stroke, or fatal stroke. However, the effect of relation was not a statistically significant, and the results were not affected by subgroup analysis.

**Table 4:** Relative risk of stroke and change in total cholesterol and triglyceride concentration between fibrates and placebo therapy

**Source**	**Triglyceride lowering**	**Total cholesterol lowering**	**Relative risk (RR) and 95% CI for stroke**	**Relative risk (RR) and 95% CI for fatal stroke**
BIP study [8] (2000)	11.0%	3.0%	0.93 (0.68, 1.27)	-
VA-HIT study [10] (1999)	31.0%	4.0%	0.73 (0.53, 1.00)	0.33 (0.09, 1.23)
Acheson J [17] (1972)	NR	6.4%	1.07 (0.70, 1.63)	0.41 (0.08, 2.00)
Veterans Administration Cooperative Study Group [18] (1973)	33.2%	3.7%	1.58 (0.97, 2.59)	0.70 (0.23, 2.19)
Coronary Drug Project Research Group [19] (1975)	22.3%	6.5%	1.11 (0.92, 1.34)	-
WHO-COOP committee of principal Investigators [20] (1978)	NR	9.0%	1.18 (0.71, 1.96)	0.99 (0.47, 2.08)
LEADER study [21,22] (2002)	23.4%	8.1%	1.23 (0.85, 1.77)	1.45 (0.62, 3.37)
E J Whitney [23] (2005)	21.5%	6.8%	0.90 (0.73, 1.12)	-
FIELD study [24] (2005)	49.8%	19.6%	0.20 (0.01, 4.15)	-
The ACCORD Study Group [25] (2010)	15.6%	NR	1.06 (0.72, 1.56)	0.50 (0.15, 1.65)

Subgroup analysis was performed for stroke, and fatal stroke. We observed that gemfibrozil therapy was associated with a statistical significant difference on the risk of stroke (RR, 0.72, 95% CI, 0.53 to 0.98, P = 0.04, Table 3). Furthermore, fibrate therapy might play a role in preventing the event of fatal stroke (RR, 0.41, 95% CI, 0.17 to 1.00, P = 0.049, Table 3), when we included the trials with Jadad score of 4. However, no other significant differences were identified between the effect of fibrate therapy and placebo based on other subset factors.

Egger’s test [16] was used to check potential publication bias. There was no evidence of publication bias for the outcomes of fatal stroke (P value for Egger’s test, 0.34). However, we observed the evidence of publication bias for stroke (P value for Egger’s test, 0.034). Subsequently, we used trim and fill methods [26] and found that the conclusion was not changed after adjusting the publication bias.

## Discussion and conclusion

The results of our study indicated that fibrate therapy has no effect on the incidence of stroke, and fatal stroke. Although VA-HIT study [10] has shown that fibrate therapy can significantly reduce the risk of stroke, this significant effect became attenuated or balanced by pooling analysis with other trials.

The inverse relation between the total cholesterol lowering and incidence of stroke indicated that there is causal relationship beween total cholesterol and the risk of stroke. Previous meta-analysis [5] indicated that statins could effectively achieve target cholesterol goals and reduce the risk of the event of stroke, which demonstrated the relation between total cholesterol and the incidence of stroke. Two previous large randomized controlled trials, VA-HIT study [10] and BIP study [8], suggest different conclusions. The VA-HIT study [10] indicated that gemfibrozil therapy significantly reduced the risk of stroke, which was consistent with our study. However, BIP study [8] suggest that although bezafibrate therapy increased the high density lipoprotein (HDL) level and decreased triglyceride level, no effect was observed on the incidence of stroke. Therefore, we carried out a comprehensive systematic review and meta-analysis to explain the possible effect of fibrate therapy on the event of stroke. Our study was based on randomized controlled trials and explored any possible correlation between fibrates therapy and the outcomes of stroke-related disease.

Our main findings are compared with the findings of previous individual randomized controlled trials and support the conclusion made by all included individual trials except for VA-HIT study [10]. No significant difference in the relative risk of stroke, or fatal stroke was reported across a wide background of high-risk participants. In our study, participants with a history of stoke, diabetes mellitus, myocardial infarction, coronary disease, lower extremity arterial disease, or high levels of cholesterol, were included. However, an unimportant heterogeneity was detected on the risk of stroke or fatal stroke for the included trials. Another important factor that may affect the results is the degree of total cholesterol lowering, although stratified analysis based on the changing of total cholesterol suggested it had no effect on the risk of stroke. However, subgroup analysis indicated that the RR of fibrate therapy on the risk of stroke ranged from 0.93 to 1.15, and fatal stroke ranged from 0.69 to 0.81 when based on baseline total cholesterol level. The reason for the suboptimal effect of fibrate therapy could be that fewer trials reported the data of stroke or fatal stroke events. Furthermore, although the information about degree of total cholesterol lowering was available, few trials reported some specific index, such as the changing of LDL. As a result, we were unable to assess the relationship between the level of reduction in some specific index and the event of stroke, or fatal stroke.

The relationship between serum total cholesterol levels, LDL and stroke were described in previous individual trials [27-30]. Epidemiological studies [31-33] also indicated that low serum level of HDL had the risk of cerebrovascular event. VA-HIT study [10] also supported this conclusion. They illustrated that gemfibrozil increased serum HDL levels by 6%, reduced triglyceride levels by 31%, and the levels of serum LDL remained same. This study indicated that gemfibrozil therapy could effectively reduce the risk of stroke. However, in BIP study [8] bezafibrate therapy increased HDL by 18%, reduced TG by 21%, and LDL by 6.5%, BIP study [8] concluded that fibrate therapy did not have an effect on the incidence of stroke. We noted that the LDL values were higher in BIP study [8] than in VA-HIT study [10], which might play an important role to lessen or counter the effect of fibrate therapy on the risk of stroke.

Although our study demonstrated no significant differences between fibrate therapy and placebo in respect of fatal stroke, when excluded WHO-COOP committee of principal Investigators study [20] and LEADER study [21,22] (two trials specifically included individuals without previous with diabetes, cardiovascular disease or stroke), a sensitivity analysis indicated that fibrates might play a role in fatal stroke (RR, 0.49, 95% CI, 0.26 to 0.93, without evidence of heterogeneity of effect). The reason for this result could be that fibrate therapy resulted in improved serum lipid profiles, which contributed an important role in the reduction of the incidence of recurrent event of stroke.

In our study, subgroup analysis illustrated that gemfibrozil therapy was associated with the reduction of risk of stroke, which was decreased by 28% (RR, 0.72, 95% CI, 0.53 to 0.98). However, these conclusions might be unreliable because only a smaller number of trials [10,21] were included in such subsets. We just gave a relative result by comparing gemfibrozil therapy with placebo and provided a synthetic and comprehensive review.

Our study also has several potential limitations. Firstly, the result was based on published data, while individual patient data and original data were not available, which limited the capacity to fully explore the effects in subgroup analysis. Secondly, although subgroup analysis indicated that fibrate therapy significantly reduced the risk of stroke, or fatal stroke for patients used gemfibrozil, or Jadad score of 4, these results might be variable due to the small number of trials. Furthermore, different baseline characteristic among participants might contribute to the lack of difference revealed in our analysis.

In conclusion, our research suggested that fibrate therapy had no significant effect on stroke, or fatal stroke. Furthermore, our study could help personally appropriate judgments about their own use of fibrate therapy, and provide evidence to prevent the incidence of fatal stroke in patients with cardiovascular disease or previous stroke. Therefore, in future study, it is important to focus on patients with previous stroke or cardiovascular disease for secondary prevention of the event of stroke or recurrent stroke, and to combine other lipid-lowering therapy, such as statins, to provide an optimal therapy to prevent the incidence of stroke. We suggest that the future trials could be improved by the following ways: (i) promising interventions should be tested, including dosage, duration of treatment or combination with influencing factors, through which we might confirm the optimal time of treatment, the optimal dosage and the optimal therapy. (ii) adverse event of trials should be reported in details so that the side-effects of any treatment could be evaluated in future trials.

## Competing interests

The authors declare that they have no competing interests.

## Authors’ contributions

YHZ and JH participated in the conceptualization and design of the review, performed the selection of studies, data-extraction and -analysis, and drafted the review. YHZ and XFY were involved in the conceptualization and design of the review, and the data analysis. FFY and XZ participated in the selection of studies and data-extraction. YHZ and JL carried out the statistical analysis and interpretation of data. All authors participated in revising the manuscript and the final approval of the manuscript.

## Pre-publication history

The pre-publication history for this paper can be accessed here:

http://www.biomedcentral.com/1471-2377/13/1/prepub

## Supplementary Material

Additional file 1 PRISMA 2009 Checklist. Click here for file
